# Quality of life at work for health professionals during the COVID-19 pandemic

**DOI:** 10.1590/0034-7167-2023-0461

**Published:** 2024-06-28

**Authors:** Monalisa Maria Leandro da Silva Oliveira, Gabriela Ferreira de Oliveira Butrico, Vanessa da Silva Carvalho Vila, Katarinne Lima Moraes, Marina Aleixo Diniz Rezende, Laidilce Teles Zatta Santos, Larissa Silva Magalhães, Sergiane Bisinoto Alves

**Affiliations:** I Pontifícia Universidade Católica de Goiás. Goiânia, Goiás, Brazil; II Universidade de Brasília. Brasília, Brasília, Brazil; III Unidade Federal de Goiás. Goiânia, Goiás, Brazil

**Keywords:** Working Conditions, Occupational Health, Quality of Life, Occupational Stress, Psychological Burnout, Condiciones de Trabajo, Salud Laboral, Calidad de Vida, Estrés Laboral, Agotamiento Psicológico

## Abstract

**Objective:**

to evaluate the quality of life at work of health professionals in direct and indirect care of COVID-19 cases.

**Methods:**

this was a cross-sectional study with 156 health professionals from a referral hospital. The relationship between sociodemographic and work-related variables and perceived stress and domains of the Quality of Life at Work Scale was investigated using inferential statistics and regression.

**Results:**

Satisfaction with Compassion was moderate (mean: 38.2), with low perception of stress, Burnout and Secondary Traumatic Stress (means: 18.8, 21.6 and 19.1). There were associations between: education, salary, multiple jobs and direct care with Compassion Satisfaction; low income, being a nurse and working overtime with Burnout; and working more than 12 hours, underlying disease and hospitalization for COVID-19 with Secondary Traumatic Stress.

**Conclusion:**

quality of life at work was satisfactory, despite the presence of Burnout and Secondary Traumatic Stress.

## RESUMEN

**Objetivo:**

evaluar la calidad de vida laboral de los profesionales sanitarios que prestan atención directa e indirecta a los casos de COVID-19.

**Métodos:**

se trata de un estudio transversal llevado a cabo con 156 profesionales sanitarios de un hospital de referencia. Se investigó la relación entre las variables sociodemográficas, laborales y el estrés percibido y los dominios de la Escala de Evaluación de la Calidad de Vida Laboral mediante estadística inferencial y de regresión.

**Resultados:**

la satisfacción por Compasión fue moderada (media: 38,2), con nivel de estrés percibido, Burnout y Estrés Traumático Secundario bajos (medias: 18,8, 21,6 y 19,1). Hubo asociaciones entre: escolaridad, sueldo, pluriempleo y atención directa con la Satisfacción por Compasión; bajos ingresos, ser enfermero y hacer horas extras con el Burnout; y trabajar más de 12 horas, enfermedad subyacente y hospitalización por COVID-19 con Estrés Traumático Secundario.

**Conclusión:**

la calidad de vida en el trabajo fue satisfactoria, a pesar de la presencia de Burnout y Estrés Traumático Secundario.

## INTRODUCTION

The COVID-19 pandemic has triggered a global health crisis, which has had a profound and immediate impact on health professionals^(1)^. These workers faced intense exposure, especially during the initial phase of the pandemic, characterized by uncertainty, fear of the unknown and a high rate of transmissibility^(2-3)^. This situation has resulted in extremely challenging working conditions for these professionals. Health professionals have come to live with circumstances of great physical and psychological strain, contributing to the development of Burnout, a psychological syndrome of emotional exhaustion, depersonalization and reduced personal fulfillment^(4-6)^.

This heightened state of psychological distress can have significant repercussions, affecting these professionals’ ability to provide adequate care, leading to the experience of trauma in the workplace and, consequently, compromising quality of life at work (QOL)^(5)^. QOL is defined as the degree to which individuals’ personal needs are satisfied, derived from their experiences and expectations of growth, as well as recognition of the activities they perform at work^(7)^. Thus, job satisfaction emerges as a determining factor for quality of life^(8)^.

Evidence has shown that healthcare workers have experienced a range of impacts on their QOL during the pandemic, experiencing a wide range of emotions, both positive and negative^(3-6)^. Although they were recognized by the population for their heroic actions, these professionals also had to deal with the uncertainty surrounding the treatment and control of the disease, the constant fear of contamination and the overwhelming scenario of suffering associated with the pandemic^(3-6)^.

In addition, several studies have examined the effects of COVID-19 on job performance^(9-10)^. The results show that among workers, the work environment is negatively affected by insecurity, uncertainty, stress, fear of mortality and high levels of contagion during the COVID-19 pandemic.

In the study of the association of COVID-19 with variables such as fear, they showed higher levels associated with increased psychological distress, lower satisfaction, decreased health perceptions and increased turnover intention in frontline nurses^(9)^.

Similarly, fear and psychological distress has shown a more significant increase among healthcare workers compared to non-healthcare workers during the COVID-19 pandemic. Research in the field confirms that healthcare workers have represented a particularly relevant group in the context of mental health care during the COVID-19 outbreak^(10)^.

In the Brazilian scenario, the pandemic has exposed the vulnerabilities of the health system, especially with regard to the safety and effective protection of the professionals involved in providing care^(7)^. As these professionals have tried to adapt to their new lives in the context of the COVID-19 pandemic, it has become important to explore the negative effects of the pandemic on quality of life at work as a result of increasing levels of fear of COVID-19 with a negative impact on effectiveness, performance and job satisfaction.

Given the crucial importance of well-being at work and the urgent need to prevent burnout, this study was conducted to assess QOL, with a special focus on healthcare professionals. This is because these individuals are in direct and prolonged contact with patients and face highly stressful situations on a daily basis. Therefore, understanding the factors that contribute to the best work context and the effects of the pandemic on the quality of work of health professionals are critical to designing and implementing measures to meet the needs and concerns of this population.

## OBJECTIVE

To evaluate the quality of life at work of health professionals in direct and indirect care of COVID-19 cases.

## METHODS

### Ethical aspects

The study complied with national and international ethical recommendations for the development of research with human beings, in accordance with Resolution 466/2012, after authorization from the Research Ethics Committee of the responsible institution.

### Study design, period and location

Analytical cross-sectional study, following the guidelines of the STROBE tool(Strengthening the Reporting of Observational Studies in Epidemiology)^(11)^, carried out from March to April 2022, in a private hospital institution, a reference for the care of patients with COVID-19 in the metropolitan region of Goiânia, Goiás, Brazil.

### Sample, inclusion and exclusion criteria

Participants were selected using non-probabilistic convenience sampling. The inclusion criteria were: being a professional in the multidisciplinary team (regardless of professional category) and providing direct or indirect care to COVID-19 patients. Professionals absent due to vacation, sick leave, pregnancy leave or medical certificates were excluded.

### Study protocol

To collect the data, a survey was initially carried out of all the health professionals working at the institution. A total of 200 professionals were identified, 90 physicians registered as service providers and 15 physiotherapists in the outsourced service.

After the survey, a database was created containing information such as name, occupation, sector and work shift. During working hours, all the professionals present in the unit were visited in a private environment, and explanations were given about the objectives, risks and importance of the study, as well as guarantees of anonymity, data confidentiality and voluntary participation.

Once the participants had given their consent, the Informed Consent Form (ICF) was presented and the data collection instruments were made available. Participants were given enough time to complete the questionnaires, with an average completion time of 15 minutes.

Data collection involved three instruments: 1) a sociodemographic and work questionnaire created by the authors (containing the covariates: age, gender, skin color, city of residence, marital status, monthly income, schooling; do you live with someone who is a risk group for COVID-19? Do you have any illnesses? Position in the institution? Work shift at the institution? How many years of professional experience? Do you have more than one job? Sector you work in? Weekly working hours? Do you often work more than 12 hours a day? Does the institution have rules and routines? Have you provided or are you providing direct care to a suspected or confirmed COVID-19 patient? Is there a risk of COVID-19 contamination where you work? Have you ever had COVID-19? Have you been hospitalized?; 2) Perceived Stress Scale (PSS-10), as a covariate; and 3) the Quality of Life at Work Assessment Scale (ProQoL-BR) determined as the primary outcome.

The ProQoL scale was chosen because it is an instrument used worldwide to measure QOL. ProQoL-BR consists of 28 items covering three dimensions: Compassion Satisfaction (CS, items 3, 6, 12, 16, 18, 20, 22, 24, 27 and 30), Burnout (BO, items 1, 4, 8, 10, 15, 17, 19, 21 and 26) and Secondary Traumatic Stress (STS, items 2, 5, 7, 9, 11, 13, 14, 23 and 25)^(12)^. The answers are grouped on an ordinal Likert scale where 1 represents “not at all true” and 5 represents “completely true”. The data obtained was analyzed based on the recommendations set out in the Manual for the fifth version of ProQoL, which recommends quartile criteria for classifying each factor as high, moderate or low^(12)^.

The PSS-10 scale is a reduced version of the PSS-14, a widely used instrument for assessing perceived stress with 14 items, seven of which are positive and seven negative. The reduced version has six negative and four positive items, assessed on a 5-point Likert scale. The scores are obtained by adding up the items, with inverse scores for the positive items. The scores range from 0 to 40. Its validity was verified in a previous study, which identified two consistent structures through principal component analysis (eigenvalues: 4.62 and 1.05; Cronbach’s Alpha: 0.83 and 0.77, respectively). Subsequently, the authors concluded that a one-dimensional structure had better fit indices^(13)^. Therefore, the scores were not categorized as low, medium or high. Therefore, the higher scores represent a greater perception of stress.

### Analysis of results and statistics

Continuous variables were described using absolute frequency, relative frequency, mean and standard deviation, and the Kolmogorov-Smirnov test was used to verify the normality of the data. Absolute and relative frequencies were used to describe categorical variables. The scores on the ProQoL-BR and PSS-10 scales were analyzed using the mean (± SD) and median (minimum and maximum) measures. The Chi-square test was used to observe the effect of data normality. In addition, the association between the ProQoL-BR classification and the patient profile was analyzed by evaluating the standardized residuals (Posthoc) using the Bonferroni correction.

Multiple linear regression analysis (*Backward* method) was carried out for the ProQoL-BR domains, considering exploratory variables with p < 0.20. The data was analyzed using institutional statistical software, adopting a significance level of 5% (p < 0.05).

## RESULTS

A total of 156 health professionals took part. Most were females (82.7%) with a mean age of 32.9 years (standard deviation: 8.7). Most participants declared themselves brown (51.3%) and lived with their spouse (58.3%). Half of the sample (50.0%) reported having higher education and an income of between 2 and 3 minimum wages (45.5%) and only 15.4% reported having some comorbidity.

The average length of professional experience was 76.1 months (SD=80.4), with a predominance of nursing technicians (59.0%) and nurses (16.0%). The majority worked a 44-hour week (66.0%), during the day (60.3%) and worked more than 12 hours a day as overtime (45.5%). Only 35.9% had another employment relationship and 97.4% reported the existence of institutional rules and routines. In the context of COVID-19, 84.6% provided direct care to patients with the disease, with 32.1% reporting a frequent risk of contamination. The majority (64.7%) had already had COVID-19 and only 4.9% needed to be hospitalized.

With regard to QoL, a moderate level of CS and low levels of BO and EST were observed. The total mean score for Perceived Stress was 18.8 (SD=5.8), indicating low levels of stress (<40) (Table 1).

**Table 1:** ProQoL- BR and PSS-10 subscale scores of health professionals, Goiânia, Goiás, Brazil, 2022

	Mean ± SD	Median (Minimum - Maximum)
ProQoL-BR		
Compassion Satisfaction (CS)	38.2 ± 7.4	39.0 (10.0 - 50.0)
Burnout (BO)	21.6 ± 5.5	22.0 (10.0 - 45.0)
Secondary Traumatic Stress (STS)	19.1 ± 6.8	18.0 (10.0 - 45.0)
PSS-10		
Total Score	18.8 ± 5.8	19.0 (5.0 - 33.0)

The association between the ProQoL-BR domains and sociodemographic, work and COVID-19 patient care data was analyzed. A statistically significant association was found between KS and schooling, with 62.5% of participants with higher education having high KS. There was also an association between low CS and low to moderate income. There was a significant difference in the association between KS and work profile. Specifically, those who worked the day shift and had other employment had high CS. In addition, 97.9% of the participants with high CS provided direct care to suspected/confirmed COVID-19 patients (Table 2).

**Table 2:** Significant associations between Compassion Satisfaction and sociodemographic, work and care variables for COVID-19 patients, Goiânia, Goiás, Brazil, 2022

Variables	Compassion satisfaction			*p*
	Low	Moderadate	High	
Education				
High School	6 (85.7)^*^	54 (53.5)	18 (37.5)	0.02
Higher Education	1 (14.3)	47 (46.5)	30 (62.5)^*^	
Income				
1 minimum wage	2 (28.6)^*^	38 (37.6)^*^	7 (14.6)	0.04
2 to 3 minimum wages	3 (42.9)	44 (43.6)	24 (50.0)	
More than 3 minimum wages	2 (28.6)	19 (18.8)	17 (35.4)	
Work Shift				
Daytime	7 (100.0)^*^	61 (60.4)	26 (54.2)	0.04
Mixed	0 (0.0)	14 (13.9)	12 (25.0)	
Evening	0 (0.0)	26 (25.7)	10 (20.8)	
Another bond				
No	6 (85.7)	70 (69.3)	24 (50.0)	0.03
Yes	1 (14.3)	31 (30.7)	24 (50.0)^*^	
Direct service to COVID-19				
No	2 (28.6)	21 (20.8)	1 (2.1)	0.01
Yes	5 (71.4)	80 (79.2)	47 (97.9)^*^	

^^*^^Posthoc.

With regard to Compassion Fatigue, represented by the BO and EST domains of the ProQoL-BR scale, significant associations were found between having a low income, being a nurse and working more than 12 hours a day and Burnout (low, moderate and moderate, respectively) and between working less than 12 hours a day and having white skin color (self-declared) and Secondary Traumatic Stress (low and moderate, respectively) (Table 3).

**Table 3:** Significant associations between Burnout and Secondary Traumatic Stress and sociodemographic and labor variables, Goiânia, Goiás, Brazil, 2022

Variable	Low	Moderadate	High	*p*
Burnout				
Income				
1 minimum wage	36 (38.7)^*^	11 (17.7)	0 (0.0)	0.02
2 to 3 minimum wages	40 (43.0)	31 (50.0)	0 (0.0)	
More than 3 minimum wages	17 (18.3)	20 (32.3)	1 (100.0)	
Profession				
Pharmacy assistant	5 (5.4)	3 (4.8)	0 (0.0)	0.01
Nurse	13 (14.0)	12 (19.4)^*^	0 (0.0)	
Physiotherapist	7 (7.5)	5 (8.1)	0 (0.0)	
Physician	5 (5.4)	4 (6.5)	0 (0.0)	
Laboratory technician	3 (3.2)	0 (0.0)	0 (0.0)	
Nursing technician	57 (61.3)	35 (56.5)	0 (0.0)	
Other	3 (3.2)	3 (4.8)^*^	1 (100.0)	
Works more than 12 hours a day				0.03
No	58 (62.4)	26 (41.9)	1 (100.0)	
Yes	35 (37.6)	36 (58.1)^*^	0 (0.0)	
Secondary Traumatic Stress				
Works more than 12 hours a day				
No	69 (59.0)^*^	14 (37.8)	2 (100.0)	0.03
Yes	48 (41.0)	23 (62.2)	0 (0.0)	
Skin color				
Yellow	8 (6.8)	0 (0.0)	2 (100.0)	0.01
White	34 (29.1)	17 (45.9)^*^	0 (0.0)	
Brown	63 (53.8)	17 (45.9)	0 (0.0)	
Black	12 (10.3)	3 (8.1)	0 (0.0)	

^^*^^Posthoc.

The predictor variables in the multiple linear regression model for CS, BO and EST are shown in Table 4. For CS, the variables gender, schooling, caring for a COVID-19 patient and having a disease had a significant weight of 15% (r2=0.15). Only income and working more than 12 hours a day were significant, explaining 3% of the variability in OR (r2=0.03). For ETS, the predictor variables were: skin color, income, presence of any illness, working more than 12 hours a day and having been hospitalized for COVID-19. Working more than 12 hours a day, having an illness and having been hospitalized with COVID-19 were statistically significant, explaining 9% of the variability in EST (r2=0.09) (Table 4).

**Table 4:** Multiple linear regression between Compassion Satisfaction, Burnout, Secondary Traumatic Stress and predictor variables of health professionals, Goiânia, Goiás, Brazil, 2022

Variables	*r^2^*	*Beta*	*t*	*p*
Compassion satisfaction				
Sex (Male)	0.15	0.13	1.74	0.04
Education (Higher)		0.19	2.57	0.01
Direct COVID care		0.29	3.84	<0.01
Has an illness (yes)		0.16	2.06	0.04
Burnout				
Income	0.03	0.07	0.86	0.39
Works 12+ hours		0.02	0.30	0.76
Secondary Traumatic Stress				
Works 12+ hours (Yes)	0.09	0.24	2.48	0.02
Has an illness (Yes)		0.12	1.24	0.04
Hospitalized for COVID-19 (Yes)		0.24	2.47	0.03

## DISCUSSION

In this study, the work experienced by health professionals was characterized by exhausting working hours, multiple employment relationships and a predominance of the day shift and overtime, similar to other studies carried out in the pandemic context^(7-8)^. The COVID-19 pandemic has resulted in the hiring of health professionals, mainly nurses and nursing technicians, who are recent graduates and often face emotional difficulties when dealing with the demands of working in challenging conditions^(3,14-15)^. The inexperience of these professionals can lead to errors in the provision of services^(16)^.

In line with the above, in a cross-sectional study involving 212 third-year nursing students at the University of Granada, Spain, the assessment of academic burnout in students with no previous clinical experience and before practical training revealed that 37.8% had high levels of burnout and that the degree of professional engagement showed an inverse relationship with burnout^(17)^. A study carried out in Greece, with 125 health professionals, revealed higher levels of Burnout in people aged 30 to 39 and among nursing professionals, a characteristic profile of the workforce that has been most demanded in the pandemic^(18)^.

In the context of the pandemic, it is also known that some professionals have had to adapt to new working models in the face of uncertainty in caring for COVID-19 patients. Studies have shown that changes in work patterns and irregular shifts have affected up to 71.2% of professionals, which can have negative impacts on well-being, sleep deprivation, increased stress and mood swings, as well as compromising the quality of care^(14,19)^. The findings reinforce that a balanced working environment is essential for the health of health professionals^(18)^.

In the present study, moderate levels of CS were found, which is consistent with findings from other studies that reported high levels of CS and low levels of BO and EST in professionals caring for COVID-19 patients^(20-23)^. In addition, we observed that the educational level of the participants also had a positive influence on the levels of CS. A previous study with nurses showed a similar result, indicating that nurses with postgraduate degrees have higher CS^(24)^. In line with this, in another study, CS, Burnout and EST were more compromised in mid-level professionals^(25)^.

Emotional exhaustion was directly influenced by work experience, interpersonal relationships and rewards in a study of 184 emergency room workers. In this study, aspects such as social support and feedback significantly predicted employee well-being and reduced the risk of burnout^(26)^. These findings reinforce the need for comprehensive support strategies to mitigate burnout among health professionals.

In this study, participants with an income of up to one minimum wage had lower scores in the KS domain, which can be explained by the presence of multiple jobs and extended working hours. On the other hand, a surprising result was the association between KS and work shift, with day shift professionals showing lower KS scores. One possible explanation is that working at night allows for more social interaction outside the work environment during the day. In addition, the presence of supervisors and the greater workload during the day may have a negative influence on CS. However, these hypotheses should be interpreted with caution. A study indicates that professionals who regularly work the night shift have higher rates of Burnout, as well as facing sleep deprivation, family problems and mood swings^(5)^.

A higher prevalence of CS was observed among health professionals who had other employment relationships. This finding, despite increasing the workload, may provide greater security for professionals. A previous study indicates that a significant proportion of health professionals have a second job, especially in the medical field^(7)^. In addition, professionals who provided direct care to COVID-19 patients had higher CS scores. This suggests that these professionals were able to maintain a satisfactory balance, despite facing exhaustion during the pandemic. Similar results were found in other international studies^(22,27)^.

Health professionals earning a minimum wage showed burnout, but at low levels. Health professionals with low salaries are more likely to develop Burnout^(28)^. There was also an association between the nursing profession and moderate Burnout, similar to the findings of other studies^(28-30)^. This can be attributed to the fact that nurses perform not only direct patient care, but also managerial activities, contributing to work overload. During the pandemic, nurses faced various stressful situations, such as a lack of personal protective equipment, increased workload, fear of contamination and a high number of deaths^(30)^.

There was also an association between moderate burnout and working more than 12 hours a day. In the institutional and pandemic context, factors such as sick leave, physical illness among staff and voluntary dismissals have resulted in the need for extra working hours to ensure adequate care. Overwork can lead to mental and physical illness, as well as being related to absenteeism, accidents at work, medical errors and exhaustion^(23,30)^.

As for the ETS domain, an association was found between working less than 12 hours a day and a low level of ETS, as well as between having white skin and a moderate level of ETS. With regard to the first finding, one possible explanation is that carrying out activities in strict and highly regulated environments in terms of safety can lead to exhaustion, even when the working day is less than 12 hours a day. As for the second association, between skin color and ETS, no studies were found in the literature to support this finding. In the Brazilian context, the scarcity of studies on race is attributed to the marked miscegenation present in the population.

A previous study, carried out remotely, assessed the QWL of 97 health professionals from various Brazilian cities who cared for COVID-19 patients. It found that those with mental illness or using medication for the nervous system had more BO and ETS. In addition, CS, BO and ETS were linked to professional recognition and appreciation^(31)^. These data reinforce the importance of valuing the work of health professionals as a way of minimizing the impact of their job on their health. In line with this, a longitudinal study carried out in the Netherlands with 173 health professionals showed that greater institutional support correlated positively with work engagement and negatively with burnout symptoms^(32)^.

It was also found that shifts lasting more than 12 hours and low salaries were factors associated with higher levels of Burnout and ETS. This can be explained by the fatigue resulting from strenuous work, the high number of deaths related to COVID-19 and the fear of contamination^(30)^. In addition, this work overload associated with low salaries predominantly affects nursing technicians. A previous study assessed CS among nursing professionals in complex care units at a Brazilian university hospital and found 17.5% of professionals with a high level of Burnout and 22.0% with a high level of ETS, with a predominance of CS in nursing technicians^(33)^.

Finally, in this study, the average level of stress, as assessed by the PSS-10 scale, was low (18.8 points). This can be attributed to the safe structure, adequate working conditions, protocols already established in the institution and vaccination of professionals during the data collection period. In the context of the COVID-19 pandemic, stress in the workplace was associated with increased workload, conflicts and additional tasks related to the pandemic and fear of contamination^(30)^. High stress can have a negative impact on workers’ physical and mental health, leading to the development of chronic diseases, substance abuse, absenteeism, burnout and poor quality of life^(30)^.

### Limitations of the study

This study has limitations, such as the heterogeneity of the sample, which may make it difficult to generalize the findings, and the cross-sectional design, which does not allow us to determine whether one variable is the cause or effect of another, only to identify associations between them. Furthermore, the study was carried out after two years of the pandemic, which may have influenced the results considering that the participants were already adapted and immunized. It would be important to compare the results with studies carried out at the beginning and end of the pandemic for a more complete analysis.

### Contributions to the field

This study contributes to the health field by demonstrating that, despite the pandemic, health professionals maintained desirable levels of job satisfaction and low levels of Burnout, Secondary Traumatic Stress and perceived stress. The presence of COVID-19 case management protocols and vaccination may have influenced these positive results. In addition, the importance of accredited hospitals in promoting the safety of professionals is highlighted. Future studies could assess the maintenance of these levels in the post-pandemic period and their impact on productivity and absenteeism at work.

## CONCLUSION

The results of the study showed that the Burnout and Secondary Traumatic Stress domains were the most affected in terms of the quality of life at work of health professionals providing direct and indirect care to COVID-19 cases. However, the professionals remained productive and satisfied with their work. Factors such as education, salary, multiple jobs and direct care of COVID-19 patients influenced the findings. Despite the limitations of the study, the results provide important insights and it is suggested that future studies be carried out to assess the post-pandemic effects on the QOL of health professionals.
