# Supplementary material for: Quality of life at work for health professionals during the COVID-19 pandemic
Source: Rev Bras Enferm. 2024 Jun 28;77(Suppl 1):e20230461. doi: 10.1590/0034-7167-2023-0461 (PMC11213535; doi:10.1590/0034-7167-2023-0461)
Supplement: Translation

# Translation: Qualidade de vida no trabalho de profissionais da saúde durante a pandemia da covid-19

**DOI:** 10.1590/0034-7167-2023-0461pt

**Authors:** Monalisa Maria Leandro da Silva Oliveira, Gabriela Ferreira de Oliveira Butrico, Vanessa da Silva Carvalho Vila, Katarinne Lima Moraes, Marina Aleixo Diniz Rezende, Laidilce Teles Zatta Santos, Larissa Silva Magalhães, Sergiane Bisinoto Alves

**Affiliations:** I Pontifícia Universidade Católica de Goiás. Goiânia, Goiás, Brasil; II Universidade de Brasília. Brasília, Brasília, Brasil; III Unidade Federal de Goiás. Goiânia, Goiás, Brasil

**Keywords:** Condições de Trabalho, Saúde Ocupacional, Qualidade de Vida no Trabalho, Estresse Ocupacional, Esgotamento Psicológico

## Abstract

**Objetivo:**

avaliar a qualidade de vida no trabalho dos profissionais da saúde em atendimento direto e indireto de casos de covid-19.

**Métodos:**

estudo transversal com 156 profissionais da saúde de um hospital de referência. Investigou-se a relação entre variáveis sociodemográficas, laborais e percepção de estresse e domínios da Escala de Avaliação da Qualidade de Vida no Trabalho, por meio de estatística inferencial e de regressão.

**Resultados:**

a Satisfação por Compaixão foi moderada (média: 38,2), com percepção de estresse, Burnout e Estresse Traumático Secundário baixos (médias: 18,8, 21,6 e 19,1). Houve associações entre: escolaridade, salário, múltiplos empregos e atendimento direto com o aspecto Satisfação por Compaixão; baixa renda, ser enfermeiro e trabalhar horas extras com Burnout; e trabalhar mais de 12 horas, doença de base e internação por covid-19 com Estresse Traumático Secundário.

**Conclusão:**

a qualidade de vida no trabalho foi satisfatória, apesar da presença de Burnout e Estresse Traumático Secundário.

## INTRODUÇÃO

A pandemia da covid-19 desencadeou uma crise sanitária de alcance global, cujo impacto se deu de forma profunda e imediata sobre os profissionais da saúde^(1)^. Esses trabalhadores enfrentaram uma exposição intensa, especialmente durante a fase inicial da pandemia, caracterizada por incertezas, pelo medo do desconhecido e por uma elevada taxa de transmissibilidade^(2-3)^. Tal contexto resultou em condições de trabalho extremamente desafiadoras para esses profissionais. Os profissionais de saúde passaram a conviver com circunstâncias de grande desgaste físico e psicológico, contribuindo para o desenvolvimento do Burnout, uma síndrome psicológica de exaustão emocional, despersonalização e redução da realização pessoal^(4-6)^.

O estado acentuado de sofrimento psicológico pode ter repercussões significativas, afetando a capacidade dos profissionais de prestar cuidados adequados, levando à vivência de traumas no ambiente de trabalho e, consequentemente, comprometendo a Qualidade de Vida no Trabalho (QVT)^(5)^. A QVT é definida como o grau de satisfação das necessidades pessoais dos indivíduos, derivado de suas experiências e expectativas de crescimento, bem como do reconhecimento das atividades que desempenham no trabalho^(7)^. Assim, a satisfação no trabalho emerge como um fator determinante para a qualidade de vida^(8)^.

Evidências mostraram que os profissionais de saúde experimentaram uma série de impactos em sua QVT durante a pandemia, vivenciando uma ampla gama de emoções, tanto positivas quanto negativas^(3-6)^. Embora tenham sido reconhecidos pela população por sua atuação heroica, os profissionais da saúde também tiveram que lidar com a incerteza em relação ao tratamento e controle da doença, o temor constante de contaminação e o cenário avassalador de sofrimento associado à pandemia^(3-6)^.

Além disso, diversos estudos examinaram os efeitos da covid-19 no desempenho profissional^(9-10)^. Os resultados mostram que entre os trabalhadores, o ambiente de trabalho é afetado negativamente pela insegurança, incerteza, estresse, medo da mortalidade e elevados níveis de contágio durante a pandemia de covid-19.

No estudo de associação da covid-19 com variáveis como medo, demonstrou-se que os níveis mais elevados se relacionam ao aumento do sofrimento psicológico, à menor satisfação, à diminuição das percepções de saúde e ao aumento da intenção de rotatividade em enfermeiros da linha de frente^(9)^.

Da mesma forma, o medo e o sofrimento psicológico demonstraram um aumento mais significativo entre os profissionais de saúde em comparação com os não profissionais de saúde durante a pandemia de covid-19. Pesquisas na área confirmam que os trabalhadores de saúde representaram um grupo particularmente relevante no contexto dos cuidados com a saúde mental durante o surto de covid-19^(10)^.

No cenário brasileiro, a pandemia expôs as vulnerabilidades do sistema de saúde, especialmente no que diz respeito à segurança e proteção efetiva dos profissionais envolvidos no cuidado^(7)^. À medida que esses profissionais tentaram adaptar-se às suas novas vidas no contexto da pandemia da covid-19, tornou-se importante explorar os efeitos negativos da pandemia na qualidade de vida no trabalho em decorrência dos níveis crescentes de medo da doença com impacto negativo na eficácia, no desempenho e na satisfação no trabalho.

Diante da importância crucial do bem-estar no trabalho e da necessidade premente de prevenir o Burnout, este estudo foi conduzido com o intuito de avaliar a QVT, com um foco especial nos profissionais de saúde. Isso porque esses indivíduos estão em contato direto e prolongado com pacientes e enfrentam diariamente situações altamente estressantes. Assim sendo, compreender os fatores que contribuem para o melhor contexto do trabalho e os efeitos da pandemia na qualidade do trabalho dos profissionais de saúde são fatores fundamentais para projetar e implementar medidas para atender às necessidades e preocupações da população.

## OBJETIVO

Avaliar a qualidade de vida no trabalho dos profissionais da saúde em atendimento direto e indireto de casos de covid-19.

## MÉTODOS

### Aspectos éticos

O estudo respeitou as recomendações éticas nacionais e internacionais para o desenvolvimento de pesquisas com seres humanos, de acordo com a Resolução nº 466/2012, após autorização do Comitê de Ética em Pesquisa da instituição responsável.

### Desenho, período e local do estudo

Estudo transversal analítico, seguindo as diretrizes da ferramenta STROBE (*Strengthening the Reporting of Observational Studies in Epidemiology*)^(11)^, realizado de março a abril de 2022, em uma instituição hospitalar privada, referência para o atendimento de pacientes com covid-19 da região metropolitana de Goiânia, Goiás, Brasil.

### Amostra, critérios de inclusão e exclusão

A seleção dos participantes ocorreu por meio da amostragem não probabilística, do tipo conveniência. Os critérios de inclusão foram: ser profissional da equipe multidisciplinar (independente da categoria profissional) e prestar atendimento, direto ou indireto, a pacientes com covid-19. Foram excluídos os profissionais ausentes por motivos de férias, licença-saúde, licença-gestação ou atestado médico.

### Protocolo do estudo

Para a coleta de dados, realizou-se, inicialmente, um levantamento de todos os profissionais da saúde que atuavam na instituição. Identificou-se um total de 200 profissionais em regime celetista, 90 médicos cadastrados como prestadores de serviço e 15 fisioterapeutas do serviço terceirizado.

Após o levantamento, foi criado um banco de dados contendo informações como: nome, ocupação, setor e turno de trabalho. Durante o horário de trabalho, todos os profissionais presentes na unidade foram visitados em um ambiente reservado, sendo fornecidas as explicações sobre os objetivos, riscos e importância do estudo, bem como garantias de anonimato, confidencialidade dos dados e participação voluntária.

Uma vez obtido o consentimento dos participantes, apresentou-se o Termo de Consentimento Livre e Esclarecido (TCLE) e disponibilizou-se os instrumentos de coleta de dados. Foi concedido tempo suficiente para que os participantes respondessem aos questionários, com um tempo médio de preenchimento de 15 minutos.

A coleta de dados envolveu três instrumentos: 1) questionário sociodemográfico e laboral criado pelos autores (contendo as covariáveis: idade, sexo, cor da pele, cidade que mora, estado civil, renda mensal, escolaridade; mora com alguém que seja grupo de risco para covid-19? Você possui alguma doença? Função na instituição? Turno de trabalho na instituição? Quantos anos de experiência profissional? Possui mais de um vínculo empregatício? Setor que trabalha? Carga horaria semanal trabalhada? Você frequentemente trabalha mais que 12 horas diárias? A instituição possui normas e rotinas? Você prestou ou presta atendimento direto a paciente suspeito ou confirmado de covid-19? No local onde você trabalha, há risco de contaminação pela covid-19? Você já teve covid-19? Você ficou internado?; 2) Escala de Percepção de Estresse (PSS-10), como covariável; e 3) a Escala de Avaliação da Qualidade de Vida no Trabalho (ProQoL-BR) determinada como desfecho primário.

A escala ProQoL foi escolhida por ser um instrumento utilizado mundialmente para medir a QVT, sendo a ProQoL-BR composta por 28 itens que abrangem três dimensões: Satisfação por Compaixão (SC; itens 3, 6, 12, 16, 18, 20, 22, 24, 27 e 30), Burnout (BO; itens 1, 4, 8, 10, 15, 17, 19, 21 e 26) e Estresse Traumático Secundário (EST; itens 2, 5, 7, 9, 11, 13, 14, 23 e 25)^(12)^. As respostas são agrupadas em uma escala Likert ordinal em que 1 representa “nada verdadeiro” e 5 representa “completamente verdadeiro”. Os dados obtidos foram analisados a partir das orientações dispostas no Manual da quinta versão do ProQoL, que recomenda critérios de quartis para classificar cada fator como alto, moderado ou baixo^(12)^.

A escala PSS-10 é uma versão reduzida da PSS-14, um instrumento amplamente utilizado para avaliar a percepção do estresse com 14 itens, sendo sete positivos e sete negativos. A versão reduzida possui seis itens negativos e quatro positivos, avaliados em uma escala Likert de 5 pontos. Os escores são obtidos somando-se os itens, com pontuações inversas para os itens positivos. Os resultados variam de 0 a 40. Sua validade foi verificada em um estudo anterior, o qual identificou duas estruturas consistentes por meio da análise de componentes principais (autovalores: 4,62 e 1,05; Alpha de Cronbach: 0,83 e 0,77, respectivamente). Posteriormente, os autores concluíram que uma estrutura unidimensional tinha melhores índices de ajuste^(13)^. Dessa forma, não foi realizada categorização dos escores em baixo, médio e alto. Portanto, as maiores pontuações representam maior percepção de estresse.

### Análise dos resultados e estatística

As variáveis contínuas foram descritas por meio de frequência absoluta, frequência relativa, média e desvio padrão, verificando a normalidade dos dados pelo teste de Kolmogorov-Smirnov. As frequências absoluta e relativa foram utilizadas para a descrição das variáveis categóricas. Os escores das escalas ProQoL-BR e PSS-10 foram analisados utilizando-se as medidas média (± DP) e mediana (mínimo e máximo). Para observar o efeito de normalidade dos dados, foi empregado o teste do Qui-quadrado. Além disso, a associação entre a classificação do ProQoL-BR e o perfil dos pacientes foi analisada por meio da avaliação dos resíduos padronizados (Posthoc) com a aplicação da correção de Bonferroni.

Realizou-se a análise de regressão linear múltipla (método *Backward*) para os domínios do ProQoL-BR, considerando variáveis exploratórias com p < 0,20. Os dados foram analisados com software estatístico institucional, adotando nível de significância de 5% (p < 0,05).

## RESULTADOS

Participaram 156 profissionais da saúde. Houve predomínio do sexo feminino (82,7%) com média de idade de 32,9 anos (desvio-padrão: 8,7). A maioria dos participantes se autodeclarou parda (51,3%) e vivia com o cônjuge (58,3%). Metade da amostra (50,0%) relatou ter ensino superior e renda entre 2 e 3 salários-mínimos (45,5%) e somente 15,4% relataram ter alguma comorbidade.

A média de tempo de experiência profissional foi de 76,1 meses (DP=80,4), com predominância de técnicos em enfermagem (59,0%) e enfermeiros (16,0%). A maioria trabalhava em jornadas de 44 horas semanais (66,0%), no período diurno (60,3%) e realizava mais de 12 horas diárias de trabalho como hora extra (45,5%). Apenas 35,9% tinham outro vínculo empregatício e 97,4% relataram existência de normas e rotinas institucional. No contexto da covid-19, 84,6% dos profissionais realizaram atendimento direto a pacientes com a doença, sendo que 32,1% relataram risco frequente de contaminação. A maioria (64,7%) já teve covid-19 e apenas 4,9% precisaram ser internados.

Em relação à QVT, foram observados nível de SC moderado e baixos níveis de BO e EST. O escore médio total de Percepção de Estresse foi de 18,8 (DP=5,8), indicando baixos níveis de estresse (<40) (Tabela 1).

**Tabela 1:** Escores das subescalas de ProQoL- BR e PSS-10 dos profissionais da saúde, Goiânia, Goiás, Brasil, 2022

	Média ± DP	Mediana (Mínimo - Máximo)
ProQoL-BR		
Satisfação por Compaixão (SC)	38,2 ± 7,4	39,0 (10,0 - 50,0)
Burnout (BO)	21,6 ± 5,5	22,0 (10,0 - 45,0)
Estresse Traumático Secundário (EST)	19,1 ± 6,8	18,0 (10,0 - 45,0)
PSS-10		
Escore total	18,8 ± 5,8	19,0 (5,0 - 33,0)

Analisou-se a associação dos domínios da ProQoL-BR com dados sociodemográficos, laborais e de atendimento aos pacientes com covid-19. Foi encontrada associação estatisticamente significativa entre a Satisfação por Compaixão (SC) e a escolaridade, com 62,5% dos participantes com nível superior apresentando alta SC. Também verificou-se associação entre baixa SC e renda baixa a moderada. Houve diferença significativa na associação entre SC e o perfil laboral. Especificamente, aqueles que trabalhavam no turno diurno e tinham outro vínculo empregatício apresentaram alta SC. Ademais, 97,9% dos participantes com alta SC realizavam atendimento direto a pacientes suspeitos/confirmados de covid-19 (Tabela 2).

**Tabela 2:** Associações significativas entre Satisfação por Compaixão e variáveis sociodemográficas, laborais e de atendimento a pacientes com covid-19, Goiânia, Goiás, Brasil, 2022

Variáveis	Satisfação por Compaixão			*p*
	Baixa	Moderada	Alta	
Escolaridade				
Ensino Médio	6 (85,7)^*^	54 (53,5)	18 (37,5)	0,02
Ensino Superior	1 (14,3)	47 (46,5)	30 (62,5)^*^	
Renda				
1 salário-mínimo	2 (28,6)^*^	38 (37,6)^*^	7 (14,6)	0,04
2 a 3 salários-mínimos	3 (42,9)	44 (43,6)	24 (50,0)	
Mais de 3 salários-mínimos	2 (28,6)	19 (18,8)	17 (35,4)	
Turno				
Diurno	7 (100,0)^*^	61 (60,4)	26 (54,2)	0,04
Mista	0 (0,0)	14 (13,9)	12 (25,0)	
Noturno	0 (0,0)	26 (25,7)	10 (20,8)	
Outro vínculo				
Não	6 (85,7)	70 (69,3)	24 (50,0)	0,03
Sim	1 (14,3)	31 (30,7)	24 (50,0)^*^	
Atendimento direto à covid-19				
Não	2 (28,6)	21 (20,8)	1 (2,1)	0,01
Sim	5 (71,4)	80 (79,2)	47 (97,9)^*^	

^^*^^Posthoc.

No tocante Fadiga por Compaixão, representado pelos domínios BO e ETS da escala ProQoL-BR, foram detectadas associações significativas entre ter baixa renda, ser enfermeiro e trabalhar mais que 12 horas diárias e Burnout (baixo, moderado e moderado, respectivamente) e entre trabalhar menos que 12 horas diárias e ter cor da pele branca (autodeclarada) e Estresse Traumático Secundário (baixo e moderado, respectivamente) (Tabela 3).

**Tabela 3:** Associações significativas entre Burnout e Estresse Traumático Secundário e variáveis sociodemográficas e laborais, Goiânia, Goiás, Brasil, 2022

Variáveis	Baixo	Moderado	Alto	*p*
Burnout				
Renda				
1 salário-mínimo	36 (38,7)^*^	11 (17,7)	0 (0,0)	0,02
2 a 3 salários-mínimos	40 (43,0)	31 (50,0)	0 (0,0)	
Mais de 3 salários-mínimos	17 (18,3)	20 (32,3)	1 (100,0)	
Profissão				
Auxiliar de Farmácia	5 (5,4)	3 (4,8)	0 (0,0)	0,01
Enfermeiro	13 (14,0)	12 (19,4)^*^	0 (0,0)	
Fisioterapeuta	7 (7,5)	5 (8,1)	0 (0,0)	
Médico	5 (5,4)	4 (6,5)	0 (0,0)	
Técnico de laboratório	3 (3,2)	0 (0,0)	0 (0,0)	
Técnico em enfermagem	57 (61,3)	35 (56,5)	0 (0,0)	
Outro	3 (3,2)	3 (4,8)^*^	1 (100,0)	
Trabalha mais que 12 horas diárias				0,03
Não	58 (62,4)	26 (41,9)	1 (100,0)	
Sim	35 (37,6)	36 (58,1)^*^	0 (0,0)	
Estresse Traumático Secundário				
Trabalha mais que 12 horas diárias				
Não	69 (59,0)^*^	14 (37,8)	2 (100,0)	0,03
Sim	48 (41,0)	23 (62,2)	0 (0,0)	
Cor da pele				
Amarelo	8 (6,8)	0 (0,0)	2 (100,0)	0,01
Branco	34 (29,1)	17 (45,9)^*^	0 (0,0)	
Pardo	63 (53,8)	17 (45,9)	0 (0,0)	
Preto	12 (10,3)	3 (8,1)	0 (0,0)	

^^*^^Posthoc.

As variáveis preditoras no modelo de regressão linear múltipla para SC, BO e ETS estão expostas na Tabela 4. Para a SC, as variáveis sexo, escolaridade, atender paciente com covid-19, ter alguma doença apresentaram peso significativo de 15% (r^2^=0,15). Apenas renda e trabalhar mais de 12 horas diárias apresentaram peso significativo, explicando 3% da variabilidade no BO (r^2^=0,03). Já para ETS, as variáveis preditoras foram: cor da pele, renda, presença de alguma doença, trabalhar mais de 12 horas diárias e ter sido internado por covid-19. Trabalhar mais de 12 horas diárias, ter alguma doença e ter sido internado com covid-19 apresentaram significância estatística, explicando 9% da variabilidade no ETS (r^2^=0,09) (Tabela 4).

**Tabela 4:** Regressão linear múltipla entre Satisfação por Compaixão, Burnout, Estresse Traumático Secundário e variáveis preditoras de profissionais da saúde, Goiânia, Goiás, Brasil, 2022

Variáveis	*r^2^*	*Beta*	*t*	*p*
Satisfação por Compaixão				
Sexo (Masculino)	0,15	0,13	1,74	0,04
Escolaridade (Superior)		0,19	2,57	0,01
Atendimento direto à COVID		0,29	3,84	<0,01
Tem doença (sim)		0,16	2,06	0,04
Burnout				
Renda	0,03	0,07	0,86	0,39
Trabalha + 12 h		0,02	0,30	0,76
Estresse Traumático Secundário				
Trabalha + 12 h (Sim)	0,09	0,24	2,48	0,02
Tem doença (Sim)		0,12	1,24	0,04
Ficou internado por covid-19 (Sim)		0,24	2,47	0,03

## DISCUSSÃO

No presente estudo, o trabalho vivenciado pelos profissionais de saúde foi caracterizado por jornadas de trabalho exaustivas, múltiplos vínculos empregatícios e predominância do turno diurno e realização de horas extras, de modo semelhante a outros estudos realizados no contexto pandêmico^(7-8)^. A pandemia da covid-19 resultou na contratação de profissionais da saúde, principalmente enfermeiros e técnicos de enfermagem, recém-formados, que muitas vezes enfrentam dificuldades emocionais ao lidar com as demandas laborais em condições desafiadoras^(3,14-15)^. A inexperiência desses profissionais pode levar a erros na prestação de serviços^(16)^.

Em consonância com o exposto acima, em um estudo transversal envolvendo 212 estudantes de enfermagem do terceiro ano na Universidade de Granada, na Espanha, a avaliação do esgotamento acadêmico em alunos sem experiência clínica prévia e antes do treinamento prático revelou que 37,8% apresentavam níveis elevados de esgotamento e que o grau de engajamento profissional apresentou uma relação inversa com o esgotamento^(17)^. Já o estudo realizado na Grécia, com 125 profissionais da saúde, revelou maiores níveis de Burnout em pessoas com faixa etária de 30 a 39 anos e entre os profissionais da enfermagem, perfil característico da força de trabalho que foi mais demandada na pandemia^(18)^.

No contexto da pandemia, sabe-se também que alguns profissionais tiveram que se adaptar a novos modelos de trabalho diante da incerteza no cuidado aos pacientes com covid-19. Estudos mostraram que mudanças nos padrões de trabalho e turnos irregulares afetaram até 71,2% dos profissionais, o que pode causar impactos negativos no bem-estar, privação de sono, aumento do estresse e alterações de humor, além de comprometer a qualidade do atendimento^(14,19)^. Os achados reforçam que um ambiente de trabalho equilibrado é essencial para a saúde dos profissionais da saúde^(18)^.

No presente estudo, níveis moderados de SC foram encontrados, o que é consistente com achados de outros estudos que relataram altos níveis de SC e baixos níveis de BO e ETS em profissionais que cuidam de pacientes com covid-19^(20-23)^. Além disso, observamos que o nível educacional dos participantes também influenciou positivamente os níveis de SC. Um estudo anterior com enfermeiros mostrou resultado semelhante, indicando que enfermeiros com pós-graduação têm maior SC^(24)^. Em consonância com outro estudo, a SC, o Burnout e o ETS tiveram maior comprometimento em profissionais de nível médio^(25)^.

A exaustão emocional foi diretamente influenciada pela experiência de trabalho, relacionamentos interpessoais e recompensas em um estudo realizado com 184 profissionais de pronto-socorro. Nesse estudo, aspectos como apoio social e feedback influenciavam significativamente o bem-estar dos funcionários e reduziam o risco de esgotamento^(26)^. Esses achados reforçam a necessidade de estratégias abrangentes de apoio para mitigar o esgotamento entre profissionais da saúde.

No presente estudo, os participantes com renda de até um salário-mínimo apresentaram pontuações mais baixas no domínio SC, o que pode ser explicado pela presença de múltiplos vínculos empregatícios e jornadas estendidas. Por outro lado, um resultado surpreendente foi a associação entre SC e o turno de trabalho, com os profissionais do turno diurno apresentando pontuações mais baixas de SC. Uma possível explicação é que o trabalho em horário noturno permite maior interação social fora do ambiente de trabalho durante o dia. Além disso, a presença de supervisores e maior demanda de trabalho durante o período diurno podem influenciar negativamente a SC. No entanto, as hipóteses devem ser interpretadas com cautela. Ademais, um outro estudo indica que profissionais que trabalham regularmente no período noturno têm maiores taxas de Burnout, além de enfrentarem privação de sono, problemas familiares e alterações de humor^(5)^.

Foi observada uma maior prevalência de SC entre os profissionais da saúde que possuíam outro vínculo empregatício. Esse achado, apesar de aumentar a carga horária de trabalho, pode proporcionar maior segurança aos profissionais. Um estudo anterior indica que uma parcela significativa de profissionais da saúde possui um segundo emprego, especialmente na área médica^(7)^. Além disso, os profissionais que prestavam atendimento direto a pacientes com covid-19 apresentaram escores mais elevados de SC. Isso sugere que esses profissionais foram capazes de manter um equilíbrio satisfatório, mesmo enfrentando exaustão durante a pandemia. Resultados semelhantes foram encontrados em outros estudos internacionais^(22,27)^.

Os profissionais da saúde que ganhavam um salário-mínimo apresentaram Burnout, porém em níveis baixos. Ainda, profissionais da saúde com baixos salários estão mais propensos a desenvolverem Burnout^(28)^. Observou-se, também, associação entre a profissão de enfermeiro e Burnout moderado, similarmente aos achados de outros estudos^(28-30)^. Isso pode ser atribuído ao fato de que os enfermeiros desempenham não apenas o cuidado direto aos pacientes, mas também atividades gerenciais, contribuindo para a sobrecarga de trabalho. Durante a pandemia, os enfermeiros enfrentaram várias situações estressantes, como falta de equipamentos de proteção individual, aumento da carga de trabalho, medo de contaminação e alto número de mortes^(30)^.

Foi observada, ainda, uma associação entre Burnout moderado e executar mais que 12 horas diárias de trabalho. No contexto institucional e pandêmico, fatores como afastamentos, doenças físicas da equipe e desligamentos voluntários resultaram na necessidade de jornadas extras para garantir a assistência adequada. O excesso de trabalho pode levar ao adoecimento mental e físico, além de estar relacionado ao absenteísmo, acidentes de trabalho, erros médicos e exaustão^(23,30)^.

Quanto ao domínio ETS, identificou-se associação entre a jornada de trabalho diária inferior a 12 horas e um nível baixo de ETS, assim como entre a pele branca e um nível moderado de ETS. Concernente ao primeiro achado, uma possível explicação é que a realização de atividades em ambientes rigorosos e altamente regulamentados em termos de segurança pode levar à exaustão, mesmo quando a jornada de trabalho é inferior a 12 horas diárias. Quanto à segunda associação, entre cor da pele e ETS, não foram encontrados na literatura estudos que sustentem o achado. No contexto brasileiro, a escassez de estudos sobre raça é atribuída à marcante miscigenação presente na população.

Um estudo anterior, realizado de forma remota, avaliou a QVT de 97 profissionais de saúde de diversas cidades brasileiras, que cuidavam de pacientes com covid-19. Constatou-se que aqueles com doenças mentais ou usando medicamentos para o sistema nervoso apresentavam mais BO e ETS. Ademais, SC, BO e ETS estiveram ligados ao reconhecimento e à valorização profissional^(31)^. Esses dados reforçam a importância da valorização do trabalho do profissional da saúde como forma de minimizar os impactos da função laboral sobre a saúde. Em concordância, um estudo longitudinal, realizado nos Países Baixos com 173 profissionais da saúde, demonstrou que um maior apoio institucional se correlacionou positivamente com o engajamento no trabalho e negativamente com sintomas de Burnout^(32)^.

Constatou-se, ainda, que turnos com duração superior a 12 horas e baixos salários foram fatores associados a maiores níveis de Burnout e ETS. Isso pode ser explicado pelo cansaço resultante de um trabalho extenuante, número elevado de óbitos relacionados à covid-19 e medo de contaminação^(30)^. Ademais, há que se considerar que a sobrecarga de trabalho associada a baixos salários afeta, predominantemente, técnicos de enfermagem. Um estudo anterior avaliou a SC entre profissionais de enfermagem em unidades de cuidados complexos de um hospital universitário brasileiro e detectou 17,5% dos profissionais com alto nível de Burnout e 22,0% com alto nível de ETS, com predominância de SC em técnicos de enfermagem^(33)^.

Por fim, no presente estudo, o nível médio de estresse, avaliado pela escala PSS-10, foi baixo (18,8 pontos). Isso pode ser atribuído à estrutura segura, condições adequadas de trabalho, protocolos já estabelecidos na instituição e vacinação dos profissionais durante o período de coleta dos dados. No contexto da pandemia da covid-19, o estresse no ambiente de trabalho esteve associado ao aumento da carga de trabalho, conflitos e tarefas adicionais relacionadas à pandemia e ao medo de contaminação^(30)^. O estresse elevado pode ter impactos negativos na saúde física e mental dos trabalhadores, levando ao desenvolvimento de doenças crônicas, uso de substâncias, absenteísmo, Burnout e baixa qualidade de vida^(30)^.

### Limitações do estudo

Este estudo tem limitações, como a heterogeneidade da amostra, que pode dificultar a generalização dos achados, e o desenho transversal, que não permite determinar se uma variável é a causa ou o efeito de outra, apenas identificar associações entre elas. Ademais, o estudo foi realizado após dois anos de pandemia, o que pode ter influenciado os resultados considerando que os participantes já estavam adaptados e imunizados. Seria importante comparar os resultados com estudos realizados no início e no fim da pandemia para uma análise mais completa.

### Contribuições para a área

Este estudo contribui para a área da saúde ao demonstrar que, apesar da pandemia, os profissionais da saúde mantiveram níveis desejáveis de satisfação no trabalho e baixos níveis de Burnout, Estresse Traumático Secundário e estresse percebido. A presença de protocolos de manejo de casos de covid-19 e a vacinação podem ter influenciado nos resultados positivos. Além disso, destaca-se a importância dos hospitais acreditados na promoção da segurança dos profissionais. Estudos futuros podem avaliar a manutenção desses níveis no período pós-pandemia e seu impacto na produtividade e no absenteísmo no trabalho.

## CONCLUSÕES

Os resultados do estudo evidenciaram que os domínios Burnout e Estresse Traumático Secundário foram os mais afetados concernentes à qualidade de vida no trabalho de profissionais da saúde em atendimento direto e indireto a casos de covid-19. Porém, os profissionais mantiveram-se produtivos e satisfeitos com o seu trabalho. Fatores como escolaridade, salário, múltiplos empregos e atendimento direto a pacientes com covid-19 influenciaram os achados. Apesar das limitações do estudo, os resultados fornecem insights importantes, sendo assim, sugere-se a realização de estudos futuros para avaliar os desdobramentos pós-pandemia na QVT dos profissionais da saúde.
